# Practical Observations on Puerperal Fever

**Published:** 1855-07

**Authors:** L. Shanks

**Affiliations:** Professor of Obstetrics and Diseases of Females in the Memphis Medical College


### DEPARTMENT OF SELECTIONS.

### PRACTICAL OBSERVATIONS ON PUERPERAL FEVER.

BY L. SHANKS, M. D.,

Professor of Obstetrics and Diseases of Females in the Memphis Medical College.

In the November number of the Recorder, some general views were presented on the different varieties and types of Puerperal Fever. These practical views resulted not only from many years' observation of the disease in the valley of Virginia, and in this city, and the contiguous country, but from careful and extended research into the history and description of the disease and its treatment, as given by all the prominent writers on the subject.

In the article referred to, the position was assumed, that Puerperal Fever may be so modified by various and numerous causes as to present the different varieties and types, not only requiring a difference in its treatment, but presenting a vast difference in the results of its amenability to treatment of any kind, in the different varieties and types. To this disparity in the pathological nature, and form of the disease, in different sporadic cases, and in different endemics and epidemics, may be attributed, in a great degree, the difference in the success of physicians of equal learning and skill in its treatment, as is shown by their own able and faithful reports.

These liberal views are not mere professions, or exhibitions of prosessional charity and courtesy, but they are the products of much experience, investigation, and reflection upon this subject. While they fully recognise and accredit, not only the faithfulness of the description of the different varieties and types of the disease, and the skill of its treatment, as given by most of the prominent writers on the subject; they leave but little scope, for approbation, or tolerance of the opinions of the few writers who maintain, like Professor Meigs, that this disease is always, nothing more nor less, than simple inflammation in the puerperal state, with its sympathetic fever, and always requires, and is curable by copious venesection, and other evacuant treatment.

The putting forth of any specific remedy or special mode of treatment, for the infallible cure of all cases of this disease, is not promised, in the practical observations proposed to be submitted to the profession of the South-west; but rather to invite their attention more especially to this important subject, for the purpose of eliciting a comparison of views and practice among ourselves in this region of country; whereby a proper discrimination of the varieties and types of this disease, and its treatment, may be more correctly made, from the practical observation and study of its symptoms, pathology, and treatment, by the intelligent physicians of the South and West, than from the speculative notions, or the practical observations of the disease, of writers, whose range of experience has been in quite a different latitude and climate, and confined to cases resulting from, or modified by causes different from those which are mainly operative among us.

On this important medical subject then, as well as all others, we only propose to the physician of the South and West to bring forward in the medical journals, their professional views and observations, matured and perfected by all the aid they can obtain, from the most thorough research into the writings of others, and present them as offerings upon the great altar of professional literature, where all that is valuable may be saved, as by the fire of criticism, and not only afford present benefit, but be garnered and preserved in the great store-house of medical knowledge for future good.

Whilst I cannot, like Drs Clark, Denman, Hulme, Leake, White, Collins, Tennon, Gordon, Hey, Armstrong, Hamilton, Dewees, Campbell, Gooch, Ferguson, Tonnelle, Lee, Klein, Churchill, Hall, Meigs, and many others, write upon this subject from large experience of the disease, in its prevalent endemic or epidemic form, I shall endeavor to describe the varieties, and types of puerperal fever, as it has presented itself to my observation, in the occasional sporadic cases, which have occurred in many years of pretty wide extended practice.

If in the description of the disease, and the treatment, which I have found most successful, there may be anything valuable; or if it may be the means of inducing others to present from their observations and practical experience anything valuable to the profession, my object will be attained.

In the previous article, I proposed, on account of their practical importance, the following five varieties of Puerperal Fever:

1st. Simple inflammation of the uterus, its appendages, and the peritoneum.

2nd. Puerperal fever produced, and modified by malarial influences.

3rd. Inflammation of the veins and absorbent vessel extending from the uteris—constituting the phlebitic form.

4th. Puerperal fever from epidemic or contagious erysipelas.

5th. Puerperal fever produced, or modified by epidemic typhus influence.

This arrangement of the different varieties is made, not so much in reference to the parts involved in the local affection, as to the pathological nature, or character of the local and constitutional disease. Of these five varieties, the three first will be more especially considered, as I have had no personal experience of the two last, and have added them as the two varieties so fully and accurately described by many writers, as constituting the epidemic, and most fatal forms of the disease ; though these varieties have been found in many cases complicated with, or presenting a mixed, or conjoint phlebitic fever.

In the first variety of puerperal fever, is embraced all the cases of the disease, which consists of *simple* inflammation of the uterus, its appendages and the peritoneum. *Simple* inflammation of the uterus alone, or of the ovaries, or laterial ligaments alone, without extending to the peritoneum, seldom, if ever, produces the sympathetic fever and constitutional disturbance entitled to be regarded as puerperal fever. Simple inflammation of these organs, however, is liable in its progress to extend to the peritoneum, and thus to develope a case, of not merely metritis, or ovaritis, but of childbed fever, with its dangerous consequences, if not promptly arrested.

This is especially so, when the attack occurs within the first week after delivery. When from any of the exciting causes, inflammation of the uterus is produced the third, or fourth week after delivery, there is much less tendency to the dangerous complication of puerperal peritonitis with it. In these cases, however, if the metritis is not speedily relieved, the inflammation is very liable to involve the sub-celular areolar tissue contiguous to the sides of the womb, between the folds of the peritoneum, constituting the lateral ligaments, on one or both sides, and sometimes one or both ovaries.

Metritis seldom results in suppuration, but tends to terminate by resolution, or in a protracted sub-acute inflammatory engorgement and swelling of the organ, attended with more or less sanguineous discharge, and results in partial tumefaction, or morbid sensibility of a portion of its walls, most frequently its lower and posterior segment. This partial chronic inflammation of the body of the uterus is doubtless the pathological state, in the obstinate cases of uterine disease, described by Gooch, Dewees and others as irritable uterus. Instead of this result of acute metritis, the inflammation may become chronic in the cervix or os, producing induration and ulceration there, as a principal affection, or a complication of the other.

When the contained organs, or celular tissue of the lateral ligament become inflamed the third or fourth week after delivery, the tendency is not so much to puerperal peritonitis, as to circumscribed suppurative form of inflammatory action. This tendency to suppuration and abscess in the areola tissue of the lateral ligaments, is so great, that it is almost a certain result of inflammation there if it is not properly subdued. From careful attention to this subject, I am convinced that abscess of the lateral ligaments, one or both, involving sometimes the ovaries, is a much more common affection than is generally supposed. It is not confined to the puerperal state, at term, but often occurs after abortions at any period of pregnancy, and sometimes from imprudence during the menstrual period. It is not a fatal disease, except when complicated with puerperal peritonitis, which does not often take place, except when it occurs within a few days after delivery. Being distressingly painful, however, and liable to frequent periodical recurrence, or exacerbations of the symptoms, I propose in the course of these numbers to give this form of disease, about which but little has been written in the systematic works, the prominence and importance, which it deserves, so far as I can do so, by giving the result of my observations and experience in its symptoms, progress, proper treatment, &c., illustrated by cases.

Impressed with the practical importance of a correct and certain diagnosis of puerperal fever, portending rapid progress, and a fatal termination, if not properly arrested by efficient treatment, I have been led to this digressive reference to simple uterine inflammation, and the circumstances under which, it is not very liable to extend to the peritoneum generally, so as to constitute a case of puerperal fever, and therefore though not portending so much immediate danger, yet requiring vigilence and prompt and efficient treatment, to the extent necessary to secure resolution, and prevent the protracted results alluded to.

The views so far expressed, in reference to the *simple* inflammatory variety of puerperal fever, fully concur with the doctrine of Dewees and others, who maintain that child-bed fever is *puerperal peritonitis complicating metritis, and not simple metritis;* but in other varieties—*the phlebitic and the typhoid*, puerperal fever, in a fatal form may occur, without the peritoneum being seriously, if at all involved. This has been *fully* demonstrated by the symptoms, and by the autopsy of many such cases, as shown by the reports of Tonnelle, Lee, and others.

These brief preliminary views must suffice for the exposition of the positions assumed as to the forms, or varieties of puerperal fever, in a practical point of view. The object proposed, being not so much a discussion of debateable questions, in reference to its local or general pathology, as the presentation of the varieties of the disease—their characteristic symptoms, progress and treatment, by a description of the sporadic cases of its different forms which have occurred in my own practice.

The first variety then, is defined to be simple inflammation of the uterus, involving also the peritoneum, and often the sub-peritoneal pelvic organs and tissues—the ovaries and their adjacent areolar tissue.

It is needless to occupy space, by a particular description of the predisposition of these organs and tissues to disease, immediately after delivery. The greatly increased developement of their vascular, nervous, fibrous and celular tissues, during gestation; the active nutrition necessary to this evolution; mechanical support to all these parts from the outward pressure of the contents of the womb ; the nervous and physical agitation, and contusion of parturition ; the sudden removal of internal distension and mechanical support by delivery; the congestion, and stasis of the blood in the greatly developed and unsupportable vessels after delivery ; the gaping and lacerated mouths of the uterine vessels over the placental surface ; the retained, stagnant and vitiated contents of the uterine vessels, and cavity, present a natural physiological state, readily changed by any of the exciting causes of disease into a preternatural, or pathological condition. The proper involution of these organs and tissues, especially during the first few days after delivery, involves a physiological change of action, circulation, nutrition, secretion, and excretion for its accomplishment, which requires the absolute avoidance of all the causes of general excitement, or internal irritation or congestion. This is the case in healthy females, in whom no predisposition to disease exists, other than that alluded to, arising from the physiological evolution and involution, preceeding and follow parturition.

Immediately following delivery, the natural tendency is, to the organic contraction, and the contraction of tissue of the uterus, by which its vessels and cavity are evacuated and closed: to increased action of the skin, attended with some increase of thirst, but diminution of appetite ; to diminished internal nutritive action, but increased peripheral excretion ; to continued sanguineous, sero-sanguineous and sero-purulent lochial discharge from the uterus, until its gestative developement, and placento-uterine lacerations are measurably restored to the normal state ; and after two or three days of abstinance and recumbent rest and quietude, a still more derivative action is set up in the breasts, to draw off the innervation and congestion from the internal uterine organs, and thus promote the copious secretion and excretion of milk. If these favorable and natural physiological changes are not interfered with, or prevented by inproper excitement from company, stimulating drinks, or food, exercise, anxiety, over heated and close rooms, too much covering, or exposure to cold, before the eruption of the secretion of milk ; the stomach and bowels have generally so much recovered from the torpor and derangement, frequently consequent upon gestation, as to be sufficiently active, not only to relieve themselves of their morbid contents, but also, to aid by this additional excretion, the involution of the uterine organs ; the relief of partial congestion, and to control and keep in due bounds the excitement, local and general, of lactation. These important benefits resulting from a laxative state of the bowels, the second or third day after delivery, if not obtained by abstinence and unexciting regimen, should be secured by saline and alkaline laxatives.

Bearing in mind these important puerperal changes, and the physiological process by which they are accomplished, the peculiar predisposition to internal engorgement and inflammatory disease, which exists at this time, is too obvious not to be properly estimated and guarded against by the intelligent physician. All the ordinary causes of derangement of the system, and those not especially so, under other circumstances, in this condition may develope an attack of puerperal inflammation, and result in a fatal form of fever. Of these causes may be specially mentioned, the excitement of visitors and too much company, improper exercise from getting out of bed, or lifting the child too soon, exposure to cold in such a way as to check the natural perspiration, and the lochial discharge, excess of excitement from too much heat, and from stimulating food and drinks, and intestinal irritation from constipation.

These, and other causes, before the secretion of milk is established, are liable to produce undue excitement, prevent natural lactation, and diminish or suppress the lochia. When the system is thus, much disturbed, and the tendency is to the simple inflammatory form of puerperal fever, it is always indicated by rigors—sometimes a well-marked chill, or chilliness alternated with flushes of heat. The pulse soon responds to this local, or general disease. The circulation becomes hurried. The pulse, though increasing in frequency in a few hours after the attack, from 100 to 130 and sometimes 140 to 150 beats in the minute, is small, though generally corded, or somewhat tense, imparting a thrill to the touch. This contracted irritable condition of the peripheral vessels, indicates the central congestion and inflammation which exist. Previous to, or during the chill and reaction which follows it, pain and soreness in the region of the womb are complained of, which increase as the circulation becomes more and more accelerated. The pains are continuous, and though increased at intervals, they do not intermit as the ordinary, or even violent after pains do, and are increased by pressure, while uninflammatory after pains are diminished by it. The pain and soreness at the commencement of the attack may be confined to the uterine region, but especially when it occurs within a few days after delivery, the tendency is, to the extension of the pain and soreness over the abdomen, in a few hours.

When this takes place the pain rapidly increases in severity, and is attended with so much intolerance of pressure, that the patient dreads and shrinks from the approach of the hand; and requires the bedclothes to be elevated from the abdomen. As a means of relief from suffering, she lies upon her back, with the lower extremities drawn up, so as to relax the psoas and abdominal muscles, and by the elovation of the knees, to protect the abdomen from the touch even of the bedclothes. With the increase of these symptoms, the tongue becomes coated with a whitish, slimy covering, or if much gastric or biliary disturbance preceded the attack, it is more red, or thickly covered with a brownish fur. The thirst becomes greater as the symptoms progress. When the inflammation extends rapidly so as to involve the peritoneum generally, tympanitic distension of the abdomen is soon produced, attended commonly with torpor of the bowels, and sick stomach. The distension and great tenderness of the abdomen, interfere so seriously with respiration as to prevent the proper oxygenation of the blood. The complexion becomes more or less livid, the aspect of countenance distressed, the functions of the brain and nervous system, and the general innervation more and more impaired, the pulse more and more frequent and feeble, the extremities become cold, the violent pain and exquisite soreness of the abdomen then diminishes, and percussion demonstrates nervous effusions, by the dullness which has superseded the sonorous tympanitic sound, in the dependant parts of the abdomen. The bowels now, may become relaxed, and the stools involuntary, and though some of the other symptoms may exhibit a favorable change, the experienced observer will not be thus deluded, and disappoint the friends' false hopes, but while he resorts to every means that may promise even a chance of life, he will but too constantly find his unfavorable prognosis realized by the rapid sinking into general collapse, and the inevitable dissolution of his patient.

In this brief and imperfect sketch of the symptoms. progress and termination of a case of puerperal fever of the simple inflammatory variety, some prominent symptoms are merely mentioned, which deserve a little further notice. The rapid extension of the inflammation, pain and soreness over the abdomen, soon followed by tympanitic distension, sick stomach, torpor of the bowels, and the fatal progress of the disease; but they soon mask the real pathological condition, by the depression and derangement of the organic functions. In this general inflammation of the covering of the important abdominal organs, not only the functions of these organs are involved; but the ganglionic system of nerves, also participates in the inflammation affecting its peritoneal covering.

Hence the sick stomach, the tympanitic distension of the bowels, the deranged biliary and urinary secretions, and the feeble, but frequent struggling efforts of the heart to carry on the circulation of the blood.

In this array of symptoms, constituting a case of simple inflammatory puerperal fever—metritis, though it may be the beginning of the attack, but existing alone, without involving the peritoneum, could produce only a part of the roll of symptoms and morbid actions, which make up a case of well-marked puerperal fever, tending to a fatal termination.

Within a few days after delivery, before lactation is established, metritis is very liable to be complicated with peritonitis, and as a consequence with the prominent symptoms described.

Metritis, however, frequently occurs in the second or third week, without involving the peritoneum. The portions covering the womb, or constituting the lateral ligaments, are sometimes involved. In these cases, the pain and soreness are local and circumscribed, the pulse has more force and volnme, and never becomes so frequent, seldom, if ever, exceeding 100 beats in the minute. The symptoms of gastro-enteric, and other functional derangements are less prominent, and the tendency to effusion and fatal termination are much less to be apprehended in these cases, than the termination in abscess of the lateral ligaments, if the inflammation is not cut short by resolution.

In the diagnosis then, of simple puerperal metritis, from puerperal metritis complicated with peritonitis, and in the prognosis of the probable progress and termination of these affections, the number of days after the delivery, when the attack occurs, is important to be considered, with the general and local symptoms.

The second, or malarial variety of puerperal fever, may very properly be described in connection with the simple inflammatory form of the disease. The difference in its pathology and treatment, may thus be made more prominent, and less space required, than for its separate description and treatment.

In regions of country where malarial influence exists in such a degree, as that, from ordinary exciting causes, intermittent and remittent fevers are produced, it is found that most diseases, even wounds, are modified by it, in their symptoms and progress.

Females brought to the bed of confinement, laboring under intermittent, or remittent fever, are, by their puerperal condition, peculiarly liable to the developement of peritonitis, as a complication of the existing malarial fever. If fever has not been previously developed, the exhaustion of the system, and the important changes produced by parturition, or imprudence soon afterwards, may become the exciting causes of malarial fever. This internal congestion and the vascular excitement consequent upon an attack of fever, within a few days after delivery, are very apt to develop metro-peritonitis. So that in many cases of malarial fever existing previously to, or developed after delivery, the complication of puerperal metro-peritonitis is liable to occur, and a puerperal fever of a modified variety, is characterised by its more decidedly paroxysmal form. The malarial fever being the primary disease, the metro-peritonitis being the effect—the complication of the fever; but in turn producing a sympathetic reaction, which, by the commingling of diseased action, produces a form of puerperal fever, which, in a practical point of view, is entitled to be considered as a distinct variety.

In cases of zymotic fever thus complicated by the production of metro-peritonitis, the local inflammation being secondary, is generally slower in its development, than in those cases where the inflammation is primary, and the fever sympathetic.

The first paroxysm of fever may be attended with some pain and soreness in the uterine region, which subsides during the intermission, or remission, but are increased by each subsequent paroxysm, until the local disease, is so fully developed, as to modify and change the fever, into the more continued and sympathetic type.

Now, in this second variety, the primary disease is malarial fever. As the effect of the paroxysmal internal congestion, and general febrile excitement, the metro-peritonitis is developed, and a complicated form of fever is produced. Whereas, in the first variety, the causes operating upon the uterine predisposition produce metro-peritonitis, which is the primary disease, and the fever is sympathetic, or secondary. If this pathology be correct, its practical importance and value are obvious. For while all experienced physicians may, in some degree, concur in the practice of treating simple inflammation by bleeding, no intelligent physician in the South-western country, would rely upon the lancet, as an exclusive, or even main remedy, for the cure of malarial fever. If this be a correct rule of practice, based upon, and sustained by general experience, in malarial fever, not prominently complicated with local infammation as its effects, upon what rational principles can an entirely different rule of practice be adopted, in those cases where this inflammatory complication has been developed by the fever?

Thus addressing the main remedies to the cure of the effect of the fever—the local inflammation, while its cause, the malarial fever, is overlooked and left uncured.

Deeming the pathological distinction, of these two varieties, of great practical importance, it is thus prominently presented, rather than occupy the space, by a detailed account of the symptoms, progress and terminations of these forms of the disease, all of which are so fully set forth by most of the writers on this subject,

*Treatment.*—Only an outline of the treatment of of these two varieties is proposed, for the purpose of presenting in their proper prominence, and relative importance, blood-letting, purgatives, quinine, cold fomentations to the abdomen, and murcurial alteratives, as the most efficient remedies.

In the first variety of puerperal fever, when the diagnosis is clear, that metro-peritonitis exists, with pain, and soreness, extended, and stili extending from the uterus over the abdomen, attended with febrile symptoms—frequent pulse, thirst, internal and general heat, &c. ; the lancet is agreed upon on all hands, as the remedy, of first, and greatest importance and efficiency, to be resorted to as soon as possible after the attack ; and the bleeding to be carried to the extent necessary to control the symptoms, and relieve the inflammatory action.

In determining on the use of the lancet, and the quantity of blood to be abstracted, the pulse is not so reliable a guide, as the abdominal pain, soreness and heat. The pulse may soon become, after the attack, so frequent, and small, and even feeble as to deter the inexperienced practitioner from bleeding ; especially in cases where parturient hemorrhage has occurred.

In most cases, however, before the stage of effusion and coldness of the extremities comes on, after which, bleeding is inadmissable, there is a jerking or thrilling sensation imparted to the touch, though the pulse may be very frequent and small, which indicates active inflammatory action, and the use of the lancet.

The quantity of blood necessary to be taken, must be determined by the effect, not immediately upon the pulse, but upon the pain and oppression of the system. For in the state of great internal or central congestion which exists, and the consequent withdrawal of blood from, and contraction of the peripheral vessels, the pulse and heart may be so seriously affected by the abstraction of a small amount of blood from the general circulation, as to produce symptoms of syncope, before the effect of the bleeding has extended to, and relieved the central congestion, which involves the ganglionic system of nerves, and the organic functions.

Under these circumstances then, the finger should be placed on the orifice, and the bleeding suspended until the amount of blood taken from the general circulation is supplied, by the withdrawal of as much, or more from the central congestion, and the pulse and action of the heart are restored, or improved, when the flow of blood should be continued, with the same precaution, again, and again, if necessary ; until enough is abstracted to relieve the pain and oppression, and restore a more general and equalizing circulation, by removing the central congestion, which oppresses and may speedily overwhelm the organic functions.

The rule of practice then, is not to be governed by the quantity of blood withdrawn, but by the permanent effect produced upon the system. To obtain this necessary permanent effect, may require the abstraction, slowly, or at intervals, of 20 ounces, in some cases, and 40, 50, 60 to 70 in others, depending upon the constitution, violence, and stage of the disease.

During the bleeding, or intervals, gentle diffusible stimuli, hot and stimulating pediluviæ, the patient being kept in the horizontal position, may be resorted to with advantage, to assist in equalizing the circulation, and relieving the internal congestion.

The prominent symptoms being thus suspended, and measurably relieved, to maintain the advantages thus gained over the disease, 20 grains of calomel with $\frac{1}{3}$ to $\frac{1}{2}$ a grain of morphine, and 15 to 20 grains of quinine may be given internally—the quinine to be repeated in 10 grain doses every 3 or 4 hours, with as much morphine as may be necessary to aid it in relieving, and preventing the return of local irritation, congestion, and pain.

In addition to these internal remedies, a large napkin smoothly doubled and wrung out of cold, or iced, water should be applied over the abdomen, and changed as often as necessary to keep it from becoming hot, and continued as long as it is agreeable to the patient. In two hours after giving the calomel, 3 or 4 ounces of a strong infusion of senna and super-tartrate of potassa, or some other purgative should be given, and repeated every 3 or 4 hours until an active purgative effect is produced. If the murcurial given, fails to produce bilious discharges in six or eleven hours, it should be repeated, and the other purgative continued so as to keep up a laxative state of the bowels.

The quinine given every three or four hours, in 10 grain doses, with or without an opiate, according as the pain or restlessness may require, to be continued until the system is brought fully under its influence. The necessary degree of quininism, is determined by a roaring or buzzing in the ears, the diminished frequency of the pulse, the cool perspirable state of the skin, the relief of pain and restlessness, diminished thirst and internal heat and soreness. When this favorable change is produced, it should be given then in five grain doses at intervals of four to six hours, with the laxatives, until the inflammatory action is relieved.

In cases not properly treated in the commencement of the attack, or when the treatment has not been instituted early enough to prevent the effects of inflammation—the plastic lymph and serous effusion, calomel, or blue mass should be continued in smaller doses—2 to 5 grains of calomel three or four times a day, until its alterative action, or slight ptyalism is produced, so as to relieve, by its sorbefacient influence, the effect of the inflammation.

This is a brief outline of the treatment I have found most successful in the first variety, or simple inflammatory form of puerperal fever, to be adapted of course, in the extent to which it is carried, to the stage and severity of the disease, the vigor of constitution &c. of the patient.

In two particulars this course of treatment may be considered extraordinary, and be excepted too; and therefore requires some further explanation.

First, The early, and free use of quinine in a purely inflammatory disease. Without entering into a discussion of the modus operandi, and effects of quinine upon the system, I shall assume, what most practitioners, who have carefully observed its effects in large and repeated doses, will admit, that it relieves or diminishes morbid nervous sensibility, diminishes under certain circumstances the frequency of the pulse, produces a cool, relaxed state of the skin, attended often with copious perspiration, promotes the action of other remedies by increasing the secretions and excretions generally, tends to equalize the innervation and the circulation, and thus by a febrifuge and antiphologistic sedative influence, relieves the morbid action of fever and inflammation.

Now these remedial effects are precisely what are required, after the necessary abstraction of blood, to prevent the further necessity for venesection, and to effectuate the cure of the disease.

The other exception, that may be made, is to the use of cold or iced applications to the abdomen. Though this treatment was proposed by Blandis, of Hanover, more than half a century ago, such has been the influence of popular prejudice against everything cold, internally, or externally, in the puerperal state, that though cold drinks may now be allowed, the use of ice, or a cold fomentation to the abdomen, in case of the patient's death, would be sufficient, in the opinion of a late writer, to destroy the professional reputation of any one in this country, who might put it into practice.

Notwithstanding this prejudice against the external use of cold water or ice, it is advised and put in practice, unhesitatingly by the best physician in cases of uterine hemorrhage. So much is this the case, that cold applications are commonly resorted to by nurses and crones for this puerperal fever.

If popular prejudice has been so completely overcome in one child-bed affection, by prosessional influence, why may it not, in another, where the danger is less, and the benefit much greater. During the past ten years my judgment has been so decidedly in favor of the use of cold, instead of warm stupes and poultices, to the abdomen in these cases, that I have required it to be used. The practice has not only proven the safety and great benefit of this treatment, but it has invariably been found to be so agreeable and soothing to patients as to induce them to beg for its frequent renewal, and its continuance, as long as the fever and internal heat remained. So far from any chilling or unpleasant effect resulting from it; in connection with the other remedies proposed, copious perspiration, and the re-establishment of the lochia have occurred during its continuance, from the abatement of the heat, and excitement, by which they were suspended.

In the treatment of the second, or malarial variety of the disease, I shall add but little. So much importance has been attached to the antiphlogistic, and sedative influence of quinine, in the simple inflammatory form, that but little more is required in reference to the other.

The pathological doctrine assumed is, that in the malarial variety, the fever is the primary, and the metro-peritonitis, the secondary affection. The fever and inflammation standing in the relation of cause and effect. This pathological doctrine being admitted, the rule of practice should be, to remove the cause and then the effect, or to adopt a course of treatment, calculated to relieve both. After this form of disease is fully developed, the course of treatment already described in the other variety, is applicable to it, except, that, as a general rule, less blood is required to be abstracted, and the free use of quinine is more essential, and indeed indispensible.

In this form of the disease, as has been stated, the local inflammation is generally slower in its developement, requiring several paroxysms for its full production. In these cases, even after the first symptoms of metritis have been manifested, the proper anti-periodic, quinine treatment, will relieve the fever, and with it the symptoms of puerperal metritis.

